# The prognostic role of heart rate recovery after exercise and metabolic syndrome in IgA nephropathy

**DOI:** 10.1186/s12882-021-02596-4

**Published:** 2021-11-23

**Authors:** Balázs Sági, István Késői, Tibor Vas, Botond Csiky, Judit Nagy, Tibor Kovács

**Affiliations:** 1 2nd Department of Internal Medicine and Nephrology, Diabetology Center, University of Pécs, Clinical Center Medical School, Pacsirta street 1, Pécs, 7624 Hungary; 2 Fresenius Medical Care Dialysis Center Pécs, Pécs, Hungary; 3 Internal Medicine Department, Health Center of Komló, Mining rehabilitation and Nighttime Sanatorium, Komló, Hungary

**Keywords:** Heart rate recovery, Chronic kidney disease, IgA nephropathy, Renal function, Cardiovascular risk

## Abstract

**Background:**

Cardiovascular (CV) morbidity and mortality are higher in chronic kidney disease (CKD) than in the general population. Reduced heart rate recovery (HRR) is an independent risk factor for CV disease. The aim of the study was to determine the prognostic role of HRR in a homogenous group of CKD patients.

**Methods:**

One hundred and twenty-five IgA nephropathy patients (82 male, 43 female, age 54.7 ± 13 years) with CKD stage 1–4 were investigated and followed for average 70 months. We performed a graded exercise treadmill stress test. HRR was derived from the difference of the peak heart rate and the heart rate at 1 min after exercise. Patients were divided into two groups by the mean HRR value (22.9 beats/min). The composite (CV and renal) endpoints included all-cause mortality and any CV event such as stroke, myocardial infarction, revascularisation (CV) and end-stage renal disease, renal replacement therapy (renal).

**Results:**

Patients with reduced HRR (< 23 bpm) had significantly more end point events (22/62 patients vs. 9/53 patients, *p* = 0.013) compared to the higher HRR (≥23 bpm). Of the secondary the endpoints (CV or renal separately) rate of the renal endpoint was significantly higher in the lower HRR group (*p* = 0.029), while there was no significant difference in the CV endpoint between the two HRR groups (*p* = 0.285). Independent predictors of survival were eGFR and diabetes mellitus by using Cox regression analysis. Kaplan-Meier curves showed significant differences in metabolic syndrome and non-metabolic syndrome when examined at the combined endpoints (cardiovascular and renal) or at each endpoint separately. The primary endpoint rate was increased significantly with the increased number of metabolic syndrome component (Met.sy. comp. 0 vs. Met. sy. comp. 2+, primary endpoints, *p* = 0.012).

**Conclusion:**

Our results showed that reduced HRR measured by treadmill exercise test has a predictive value for the prognosis of IgA nephropathy. The presence of metabolic syndrome may worsen the prognosis of IgA nephropathy.

## Introduction

For predicting cardiovascular (CV) risk, the treadmill exercise test seems to be a more suitable non-invasive tool than routinely assessing the functional capacity by electrocardiogram or the walking test [1, 2]. Physical exercise is associated with decrease in parasympathetic tone and increase in sympathetic activity resulting in heart rate elevation. The rate of post-exercise cardiodeceleration is used as an index of cardiac vagal reactivation. Heart rate recovery (HRR) may be a predictor of cardiovascular risk associated with the sympathetic and parasympathetic tone balance [3–5]. HRR is evaluated in the first or second minutes after the peak exercise at the end of the stress test which is a validated method [6].

The prevalence of chronic kidney disease (CKD) is high all over the world [7]. On the basis of the meta-analyses it is well known that significant number of patients with CKD die from cardiovascular events before the development of end-stage renal disease (ESRD). IgA nephropathy (IgAN) is the most common primary glomerular disease all over the world [8]. Patients with IgAN are a relatively homogenous group compared with the whole CKD population.

Previous studies had demonstrated the prognostic role of HRR in patients with various heart diseases. Reduced HRR has been defined as an indicator of mortality and sudden cardiac death in coronary artery disease, in heart failure, in left ventricular dysfunction and after coronary artery revascularisation [9–13]. In addition, renal function declines at any degree, increases the risk of CV morbidity and mortality [14, 15]. In a cross-sectional study, we have earlier shown that inverse relationship exists between HRR and renal function and this study was conducted in patients with IgA nephropathy [16]. The development of insulin resistance, dyslipidemia, hypertension and metabolic syndrome could be frequently observed in CKD was previously shown to be associated with decreased HRR [17, 18]. Therefore, the aim of the present study was to investigate the prognostic role of HRR on major CV (myocardial infarction, stroke, revascularisation, cardiac death) and renal outcomes (ESRD) in patients with IgA nephropathy with longer follow-up.

## Patients and methods

We examined 125 patients with histologically confirmed IgA nephropathy by renal biopsy, who had no known heart disease, although controlled coronary artery disease (CAD) was allowed for enrolment. Patients with left Tawara-branch block on the ECG were excluded. All patients were in eligible condition to perform the stress test. There were 82 male and 43 female patients enrolled in the cohort, with a mean age of 54.7 ± 13 years. During the first visit, traditional risk factors were also evaluated, such as hypertension, carbohydrate metabolism disorder, obesity, lipid abnormalities, smoking. The CKD-EPI formula (eGFR, ml / min / 1.73 m^2^) was used to estimate kidney function. Exclusion criteria were: severe heart failure (NYHA Stage III-IV or low ejection fraction < 35%,), stroke within 3 months, myocardial infarction, uncontrolled arrhythmia, malignancy with active treatment, severe hypertension (≥180 / 110 mmHg). Patients with ESRD (CKD stage 5), renal replacement therapy, or kidney transplantation in the history were also excluded. Participant patients were then examined every 3 to 6 months (more often as needed). At these patient visits, any adverse events since the last visit were interviewed, physical status was taken, detailed laboratory tests were performed. When it was required, in view of the complaints, further CV examinations (echocardiography, ergometry, coronarography, etc.) were also completed. On the follow-up period, 10 patients did not attend on the visits, thus their data were not analysed. Our patients did not receive any steroid therapy either parenterally or orally, before the study, or during follow-up. Beta blockers and RAAS inhibitors usage did not change during follow-up. None of the patients had to stop taking these medications. Patients were tested for glucose tolerance. Prediabetic or diabetic state were set up out the basis of international criteria. The 24-h ambulatory blood pressure measurement was measured oscillometrically (ABPM, Meditech, Hungary). Body mass index (BMI) was calculated by the standard method [19]. Definition of metabolic syndrome (MS) was stated by the World Health Organization (WHO) criteria.

Echocardiography was performed prior to the stress test in all patients to assess the left ventricular systolic function. The left ventricular systolic function was characterized by ejection fraction (LVEF), which was determined by using the Quinones formula. Patients completed asymptomatic, progressive treadmill test by the standard Bruce protocol to achieve the maximal predicted heart rate (220 minus age) [20]. The tests were performed by the same professional staff. All tests were conducted in the mornings, and patients were not allowed to smoke, or take the regular medications at least two hours before the test. Beta-blockers and nitrates were stopped at least 48 h before the test. Continuous 12-lead electrocardiographic (ECG) monitoring was performed during the test, and recorded ECG results of every single minute, including the recovery phase, were printed out. Exercise capacities were measured from the first step to the peak results and were expressed in seconds as the unit. After the peak of the exercise, there was a minimal of 1-min reduction time, the treadmill speed was 1.6 km per hour. HRR was calculated from the difference of the heart rate at the peak exercise and 1 min later. Results were analyzed off-line in the printed forms. The diagnosis of CAD was established when horizontal or descending ST segment depressions (≥ 1 mm) could be observed in two or more coherent leads. For all patients, the indication of stress test was to determine the maximal exercise capacity, and the diagnosis of suspected CAD. Written informed consent was obtained in all participants after the University ethical committee had approved the study.

### Statistical analysis

Patients were divided into two groups by the mean HRR value (23 / bpm). The composite primary endpoint of the study composed of the CV endpoint of acute myocardial infarction, intervention due to acute coronary syndrome, stroke or death for any cause, and the renal endpoint of end-stage renal failure (eGFR < 15 ml/min/1,73 m^2^), or renal replacement therapy. The CV and renal endpoints were also analysed separately as secondary endpoints.

All values are expressed as mean ± SD unless otherwise indicated. Survival rates of the two groups were examined by Mantel-Cox log-rank test. The effect of HRR on survival was evaluated by Cox regression analysis. Multivariate analysis was used to explore the factors that influence the CV events and impaired renal function. As the distribution of these parameters was normal, Spearman correlation was used to determine the relationship between renal function and HRR. Independent risk factors associated with HRR were studied by univariate and multivariate linear regression analysis.

*P* < 0.05 was considered to be statistically significant. The calculations were performed by the SPSS software version 22.0.

## Results

The baseline clinical characteristics of IgA nephropathy patients are shown in Table 1. Of the 125 patients, 82 were male, 43 were female. Blood pressure values measured by 24-h ABPM were higher in the lower HRR group (HRR < 23 beats / min). Patients were treated with angiotensin converting enzyme inhibitors (ACEI), angiotensin receptor blockers (ARBs), calcium channel blockers (CCBs), beta-blockers (BBL), and statins. The resting heart rate and the stress test exercise capacity showed significant difference between the two groups. The incidence of CAD was not differed significantly between the two groups. LVEF had no difference between the two groups, but there was a significantly higher occurrence of diastolic dysfunction in the reduced HRR group (Table 1).

**Table 1:** Baseline characteristics of IgAN

Clinical data	HRR ≥ 23	HRR < 23	P
Patients (*n* = 125)	60	65	
Man/woman (n/%)	36/24 (60/40)	46/19 (71/29)	NS
Age (year)	51.4 ± 12.5	54.7 ± 13.0	NS
Average systolic RR (Hgmm)	120.1 ± 0.6	126.7 ± 0.8	0.002
Average diastolic RR (Hgmm)	73 ± 9.6	75.7 ± 9.5	NS
24 h pulse pressure (Hgmm)	47.40 ± 7.96	51.10 ± 8.85	0.012
Diurnal index systolic (%)	10.92 ± 5.08	8.36 ± 7.72	0.020
**Metabolic parameters**			
Hypertension (n, %)	41 (68)	53 (81)	NS
BMI (kg/m2)	25.17 ± 3.8	27.7 ± 4.8	0.001
Dyslipidaemia (n, %)	24 (40)	34 (52)	NS
Carbohydrate metabolic disorder (n, %)	9 (15)	21 (32)	NS
eGFR (ml/min)	94.6 ± 29.3	78.9 ± 37.9	0.005
Duration of kidney disease (year)	10.2 ± 9.7	8.8 ± 9.1	NS
Smoking (n, %)	7 (12)	11(17)	NS
Metabolic syndrome (n, %)	13 (22)	21 (32)	NS
**Therapy**			
ACEI/ARB (n, %)	46 (77)	60 (92)	NS
BB (n, %)	12 (20)	19 (29)	NS
Statin (n, %)	16 (27)	22 (34)	NS
CCB (n, %)	9 (15)	19 (29)	NS
**Ergometry**			
Average heart rate (beat/min)	72.1 ± 8.8	76.0 ± 10.9	0.016
Stress test time (s)	608 ± 178.3	550 ± 186.5	0.039
HRR (bpm)	31.2 ± 7.4	15.6 ± 5.1	< 0.001
CAD (Positive stress test)	5 (8)	12 (18)	NS
**Echocardiographic parameters**			
LV EF (%)	62.4 ± 6.9	63.2 ± 5.9	NS
LVMI	103.53 ± 15.95	109.21 ± 21.25	NS
LVM (g)	196.8 ± 41.5	212.1 ± 38.5	NS
LVEDD (cm)	6.05 ± 6.29	5.57 ± 5.13	NS
DD (n/%)	7 (11)	17 (26)	< 0.001
E/A	1.18 ± 0.32	0.93 ± 0.30	< 0.001
**Laboratory results**			
Hb (g/dl)	13.9 ± 1.6	13.6 ± 1.7	NS
MAU (mg/day)	406 ± 578.6	504 ± 642.3	NS
HUS (umol/l)	303 ± 97.4	342 ± 84.9	0.009
Total cholesterol (mmol/l)	5.01 ± 1.28	4.93 ± 1.06	NS
HDL cholesterol (mmol/l)	1.35 ± 0.57	1.20 ± 0.31	0.029
TG (mmol/l)	1.49 ± 0.88	1.93 ± 1.21	0.012

*HRR* heat rate recovery, *RR* blood pressure, *BMI* body mass index, *eGFR* estimated glomerular filtration rate, *ACEI* angiotensin converting enzyme inhibitor, *ARB* angiotensin receptor blocker, *BB* beta blocker, *CCB* calcium channel blocker, *CAD* coronary artery disease, *LV EF* left ventricle ejection fraction, *LVMI* left ventricle mass index, *LVM* left ventricular mass, *LVEDD* left ventricular end-diastolic diameter, *DD* diastolic dysfunction, *Hb* hemoglobin, *MAU* microalbuminuria, *HUS* uric acid, *HDL* cholesterol: high-density lipoprotein cholesterol, *TG* triglyceride

**Fig. 1:**
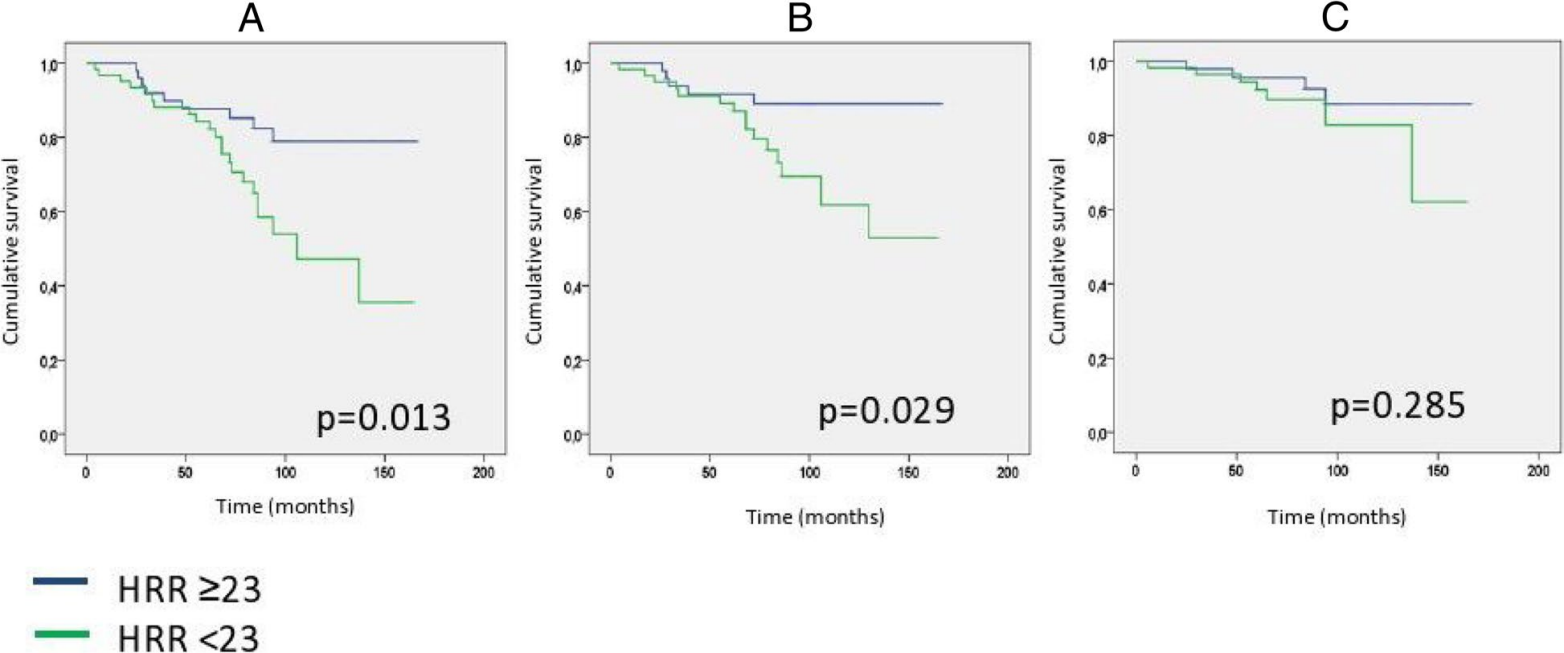
Primary combined and secondary (renal and cardiovascular) endpoints Kaplan-Meier curves based on HRR

Fig. 1A shows that the probability of the combined (CV and renal) endpoint was significantly higher in the group with reduced HRR (HRR < 23 beats / min), compared to the group with increased HRR (HRR > 23 beats / min) (Chi-square: 6.138; *p* = 0.013). For the secondary endpoints, the rate of the renal endpoint was also significantly higher in the lower HRR group (Chi-square: 4.739; *p* = 0.029) (Fig. 1B); whereas, there was no significant difference in the separate CV endpoint between the lower and the higher HRR group (Chi-square 1.145, *p* = 0.285) (Fig. 1C).

**Fig. 2:**
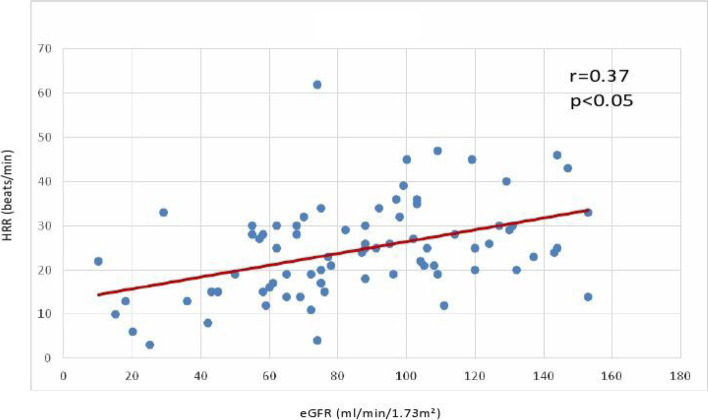
Correlation between HRR and renal function

Clinical factors that could have impact on HRR in IgA nephropathy patients were analysed in a linear regression model containing 11 confounding variables (Table 2). By the univariate test, age, all of the examined parameters (metabolic syndrome, ABPM, systolic blood pressure, ABPM systolic diurnal index, ABPM pulse pressure, BMI, dyslipidaemia, carbohydrate metabolic disorder /IFG, IGT, DM/, eGFR, ACEI/ARB, and statin therapy) were found to be associated with altered HRR (Table 2). By multivariate test, only eGFR was found to be independently associated with altered HRR (Table 2). Significant positive relationship between HRR and eGFR was observed (corr. Coeff. = 0.37) (Fig. 2).

**Table 2:** Factors associated with HRR analysed in the univariate and multivariate linear regression models

	Univariate analysis		Multivariate analysis	
Variable	R2	p	ß	p
Age	0.071	0.006	− 0.023	0.827
Metabolic syndrome	0.080	0.004	−0.181	0.411
Systolic blood pressure	0.074	0.006	0.061	0.707
Systolic diurnal index	0.049	0.027	0.114	0.252
Pulse pressure	0.054	0.021	−0.086	0.559
BMI	0.092	0.002	−0.177	0.109
Dyslipidemia	0.071	0.007	−0.110	0.264
Carbohydrate metabolic disorder	0.056	0.016	0.111	0.607
**eGFR**	**0.138**	**< 0.001**	**0.237**	**0.031**
ACEi/ARB treatment	0.090	0.002	−0.156	0.129
Statin treatment	0.046	0.030	−0.064	0.523

*BMI* body mass index, *eGFR* estimated glomerular filtration rate, *ACEI/ARB* angiotensin converting enzyme inhibitor / angiotensin receptor blocker

Significantly more metabolic risk factors, and complete metabolic syndrome occurred in the lower HRR patient group (HRR < 23 beats / min) (Table 1). The incidence of complete metabolic syndrome was 23% in the IgA nephopathy patients studied (Table 1).

In the present study, we allocated patients into two groups by the presence or absence of metabolic syndrome, and we found markedly lower HRR values in those patients who had metabolic syndrome (18.0 versus 24.5 beats per minute, *p* < 0.001).

**Fig. 3:**
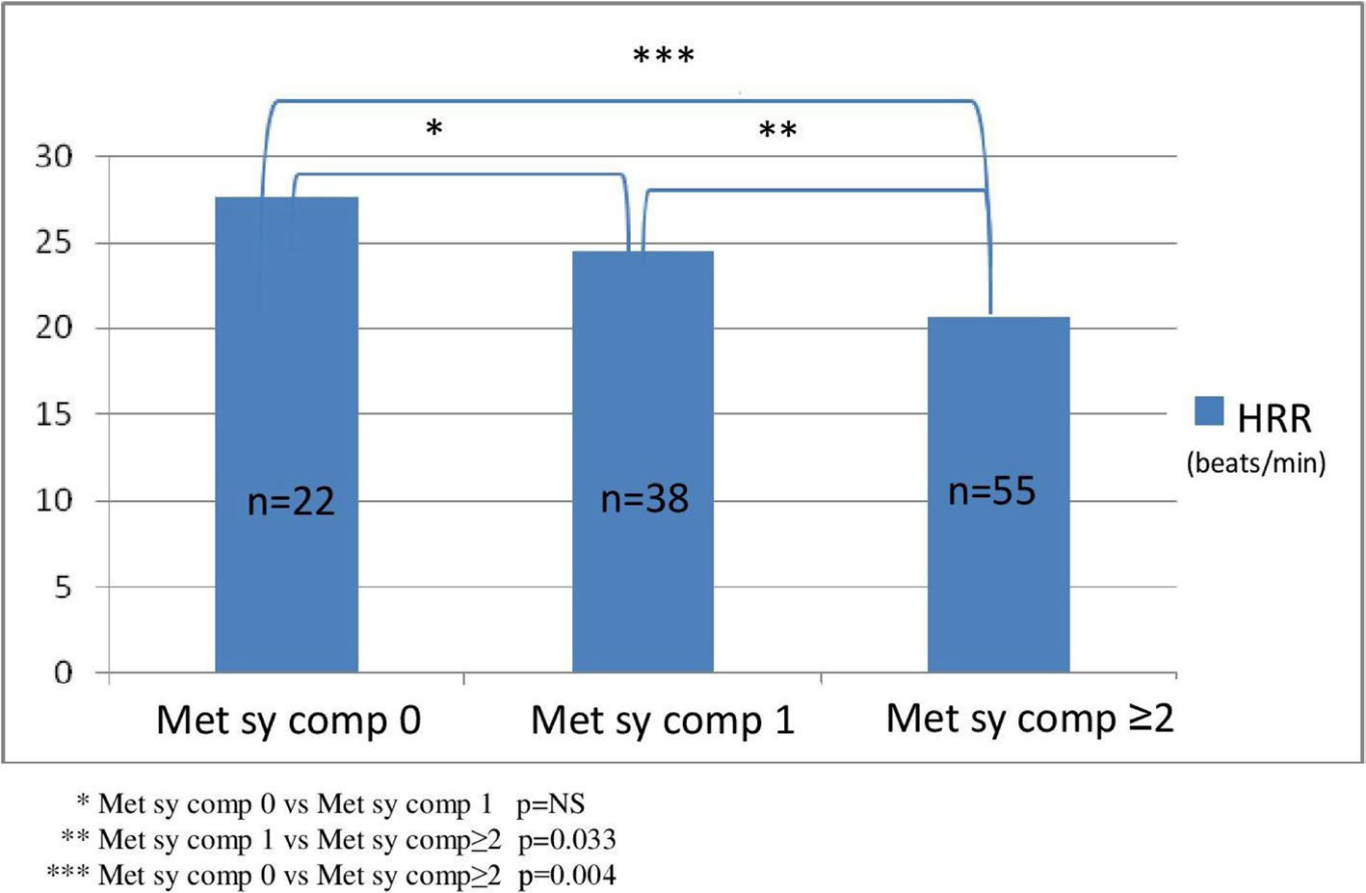
Primary combined and secondary endpoints in IgAN with or without metabolic syndrome

**Fig. 4:**
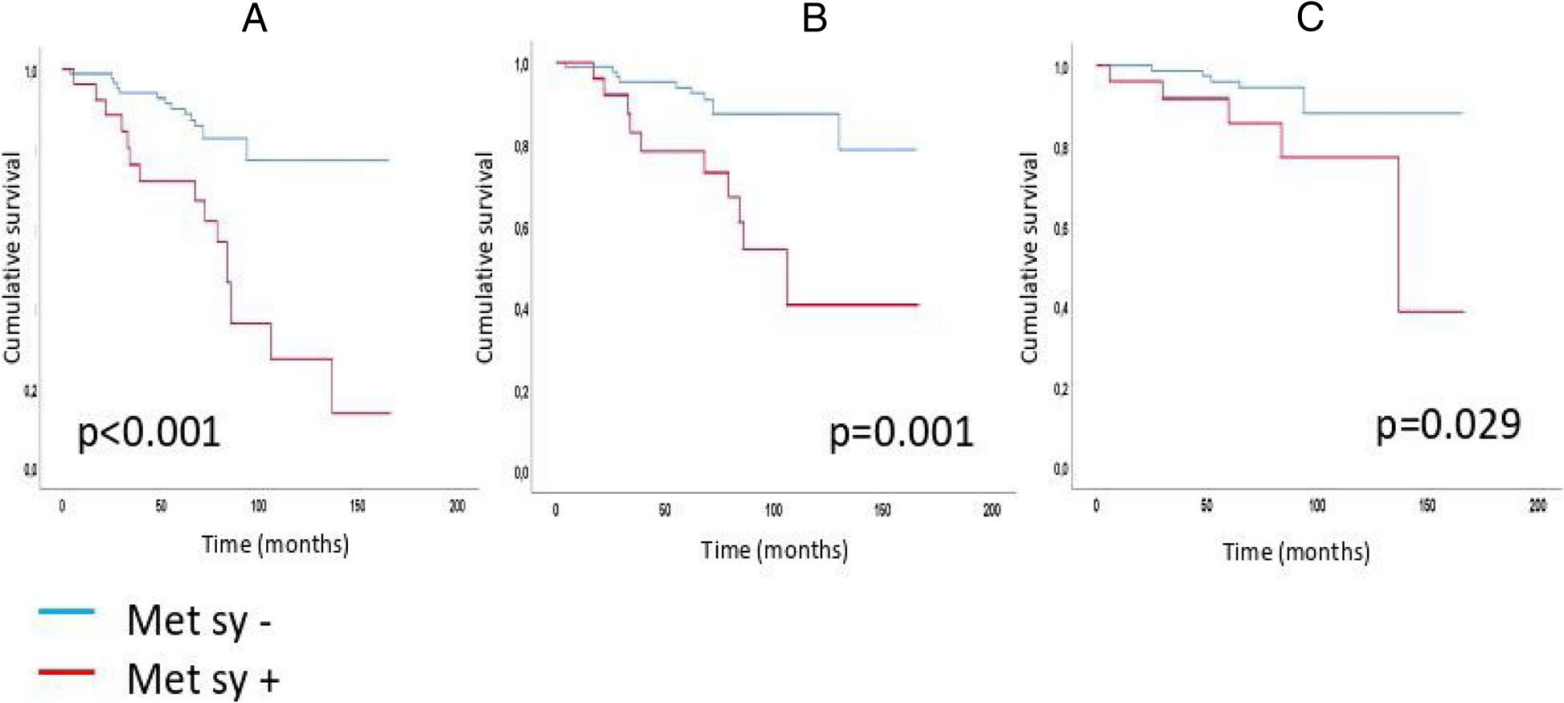
HRR values in IgAN with or without any metabolic syndrome components

**Fig. 5:**
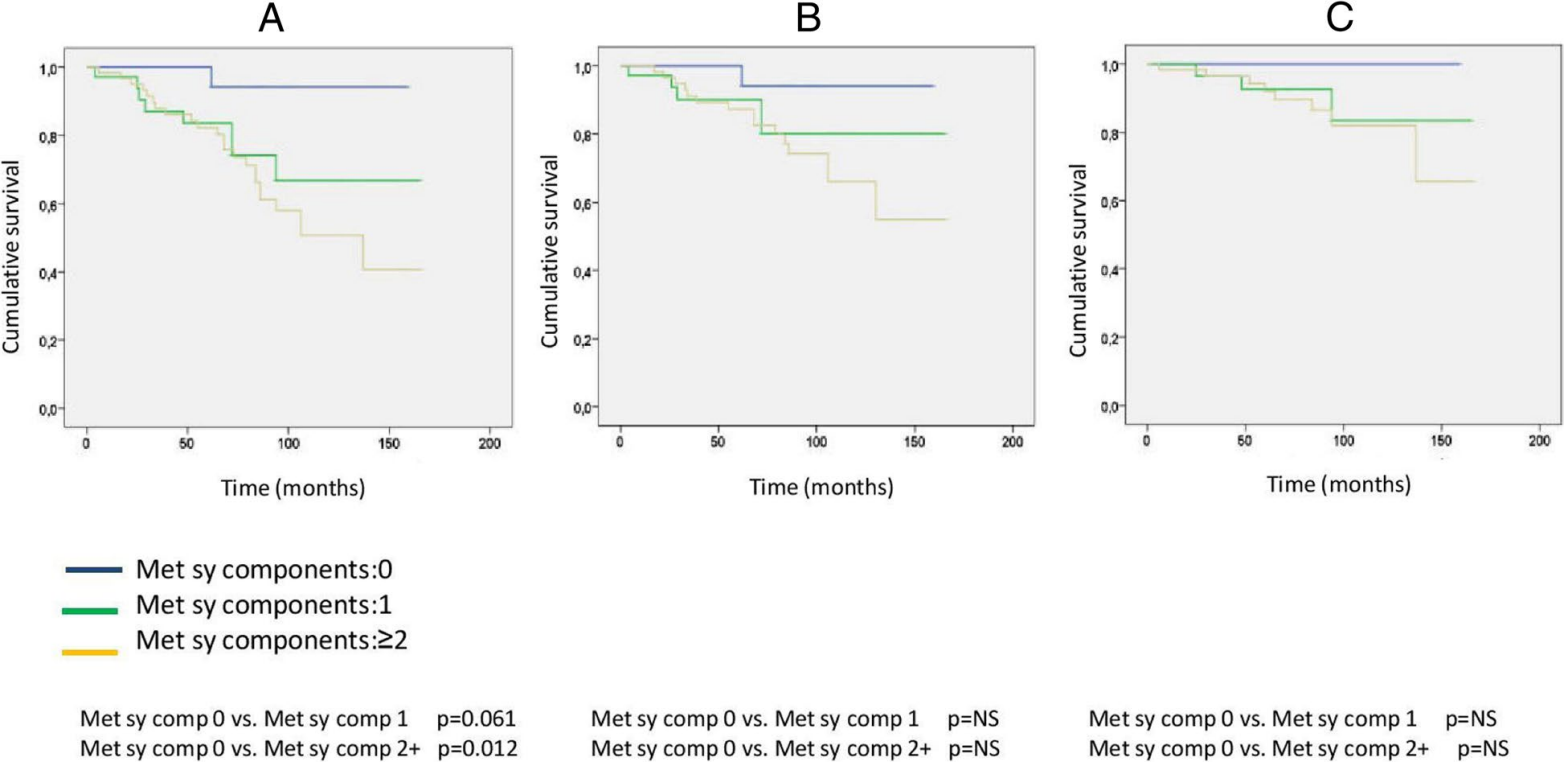
Primary combined and secondary endpoints in IgAN with or without any metabolic syndrome components

**Fig. 6:**
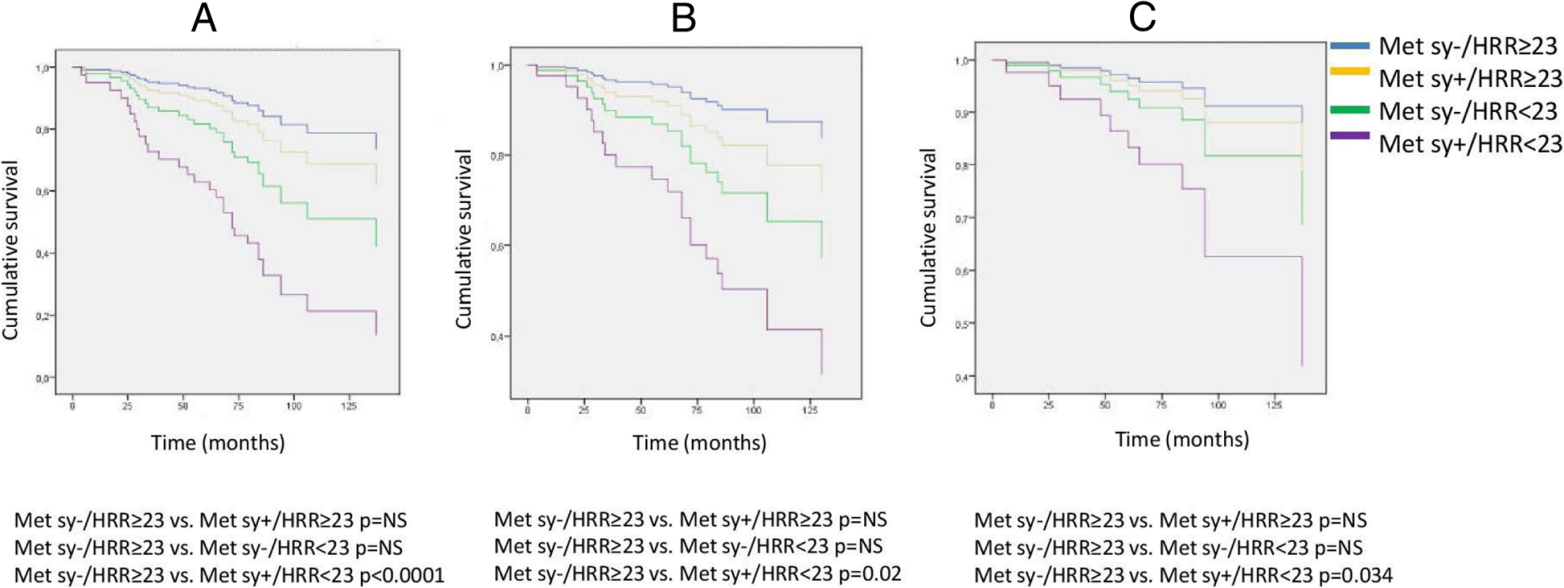
Primary combined and secondary endpoints Kaplan-Meier curves based on metabolic syndrome and HRR in different risk groups

The incidence of composite and secondary renal and CV endpoints was also significantly higher in the metabolic syndrome group (Chi-square: 18.666; p < 0.001, Chi-square: 10.692; *p* = 0.001, Chi-square: 4.778; *p* = 0.029) (Fig. 3). With an increasing number of the metabolic syndrome elements, the HRR values showed significant reductions, as well as the primary endpoint event rates had significantly increased (primary endpoint 1 versus 22 in Met Component 0 versus 2+; *p* = 0.012) as shown in Figs. 4 and 5. When we formed risk groups based on the presence or absence of metabolic syndrome and maintained or attenuated HRR, the occurrence of the primary combined and secondary renal and CV endpoints was significantly higher in the reduced HRR with metabolic syndrome group so their survival was the worst (Fig. 6). The likelihood of primary combined outcomes increased in different risk groups with the onset of metabolic syndrome and decreasing HRR (OR: 6,4) (Table 3).

**Table 3:** The primary combined and secondary renal and cardiovascular outcomes probability in a different risk groups (presence or absence of metabolic syndrome and maintained or attenuated heart rate recovery)

	Primary combined endpoints		Secondary renal endpoints		Secondary cardiovascular endpoints	
Risk groups	Odds Ratio (conf. Int.)	p	Odds Ratio (conf. Int.)	p	Odds Ratio (conf. Int.)	p
Met sy−/HRR ≥ 23	1.0	**–**	1.0	**–**	1.0	**–**
Met sy−/HRR < 23	1.6 (0.565–4.321)	0.390	1.9 (0.527–4.821)	0.332	1.4 (0.229–3.112)	0.687
Met sy+/HRR ≥ 23	2.8 (0.722–10.982)	0.136	3.1 (0.577–11.456)	0.185	2.2 (0.279–8.987)	0.494
Met sy+/HRR < 23	6.4 (2.550–16.149)	< 0.0001	6.5 (1.969–16.321)	0.002	5.1 (1.129–14.867)	0.034

*Met sy* - without metabolic syndrome, *Met sy +* with metabolic syndrome, *HRR* heart rate recovery

## Discussion

In the present follow-up study, we demonstrated that reduced HRR has prognostic significance in patients with IgAN, a relatively homogeneous group of CKD patients. We found that IgA nephropathy patients with reduced HRR values have a significantly increased rate of adverse primary CV and renal events compared to those who presented with higher HRR values. Our data confirmed again the inverse correlation between HRR and eGFR, in line with our observations in a previous cross-sectional study [16]. In case of IgA nephropathy with an increase in metabolic syndrome components and the development of complete metabolic syndrome which may be associated with autonomic dysfunction, the first sign of which may be a decrease in HRR, this phenomenon may warrant an increased CV and renal risk in these patients.

Previously, the predictive role of HRR for CV morbidity and mortality had been studied in many diseases, including CKD, heart failure, CAD, DM, Bechet’s disease, and systemic lupus erythematosus [21–23]. The relationship between reduced HRR and CV mortality is still not fully understood. However, increased susceptibility to atherosclerosis has been advocated with reduced HRR values. In addition, reduced HRR values pointed to the presence of autonomic dysfunction in the Framingham study [24], indicating a prognostic role as well.

Increased parasympathetic activity associated with reduced heart rate and blood pressure may be protective against the ischemia-induced dysrhythmias [25]. Cole et al. involving 2428 patients with no diagnosis of CAD, found that the reduced 1-min heart rate recovery after stress test indicated reduced vagal activity, and was proven as a strong predictor of all-cause mortality, independent of the resting heart rate or heart rate changes upon the test [26].

In the Lipid Research Clinics Prevalence study 2994 women, negative for CV disease were subjected to exercise test, and this long-term follow-up trial had established that reduced HRR was associated with all-cause and CV mortality [27]. Data published earlier by Cheng et al. demonstrated that reduced HRR increases the risk of mortality in diabetic patients by 1.5 to 2-fold compared to patients with the maximum level of HRR, even after corrected for several variables, including age and resting heart rate [28]. In a study of 12.712 asymptomatic, angina-free male patients Jae et al. found that the reduced HRR correlated well with the increased intima-media thickness of the carotid arteries [29]. The relationship between reduced HRR and atherosclerosis is not entirely clarified, however, it has been implicated that impaired HRR may be associated with endothelial dysfunction leading to vascular inflammation and accelerating the progression of atherosclerosis. Consequently, HRR is an independent predictor of endothelial dysfunction [30]. The autonomic dysfunction, endothelial dysfunction, and advanced atherosclerosis may be key factors in our patients with IgAN as well.

Previous studies have shown that proteinuria is associated with endothelial dysfunction [31]. Therefore, proteinuria occurring during nephrotic syndrome (NS) may also be associated with endothelial dysfunction and atherosclerosis. A Turkish research team reported reduced HRR in patients with primary NS, indicating higher CV risk in these patients [32]. A recent meta-analysis by Qiu et al. provided several putative elucidations for the adverse effects of reduced HRR on CV events and all-cause mortality, but the precise mechanism remains unknown [33]. Our results are consistent, since we showed that there was poorer functional capacity and worse exercise time in patients with reduced HRR, and also found increased number of adverse primary endpoints events in the same group of patients with reduced HRR.

Moreover, it has been recognized that intact autonomic nerve function is essential in the regulation of glucose homeostasis, as the parasympathetic fibers stimulate beta cells to release insulin in response to elevated glucose levels, in contrast, the sympathetic activity inhibits insulin secretion [34]. Autonomic dysfunction, reflected by reduced HRR, also reduces insulin secretion and increases glucose levels, leading to the development of diabetes mellitus and other long-term disorders, such as CAD via various mechanisms, including glucose toxicity, chronic inflammation, and endothelial dysfunction [35–37]. Our results suggest that high prevalence of MS in IgAN may support this hypothesis.

In addition, as higher HRR reflects parasympathetic activity and increased parasympathetic tone affords antiarrhythmic effects [38], it is likely that reduced HRR may predict mortality due to the increased risk of arrhythmia.

In the present study, we noticed considerably more primary adverse endpoints in the presence of metabolic syndrome, supporting the clinical relevance of complex metabolic risk reduction in patients with IgA nephropathy, which could draw medical attention to high-risk patients. Our results confirm that decreased HRR may be a clinical sign of metabolic imbalance caused by autonomic dysfunction (or vice versa) and all of these alterations may play a role in the progression of CKD.

According to Carreira et al., significantly lower HRR could be observed in every minute of the recovery phase in hemodialysis (HD) patients compared to the control group of nonrenal patients [39]. Slow recovery of heart rate reflects to inadequate recovery of cardiac vagal activity, hence, it could be a useful indicator of CV events both in patients with heart disease and in healthy individuals [40–42], and also a predictor of mortality, regardless of the severity of CAD described by angiography [10]. But in our study, the necessity of renal replacement therapy was defined as a renal endpoint, we investigated only CKD1–4 stage patients, and we have found similar tendency. It can be assumed that all of these factors may become increasingly important in the early declines of renal function.

Autonomous dysfunction, characterized by reduced HRR is one of the most important CV risks markers, and also an independent predictor of cardiac death in patients with CV disease. This may be indicated by decreased HRR in early CKD and may predict that patients with CKD may die in a CV event before reaching ESRD. Of note, the prognostic value of autonomic dysfunction seems as important as overt CAD per se for CV outcomes in patients with heart disease [10, 11].

McManus et al. had examined renal function and HRR in the population of patients with heart disease. They found that increased circulating cystatin C levels that indicates reduced renal function had almost a linear relationship with decreased functional capacity and HRR [43]. Our results in patients with CKD supported these findings (see Fig. 4). Increased sympathetic activity, as well as decreased parasympathetic activity could contribute to autonomous imbalance. Sympathetic hyperactivity evidently exists in CKD patients; increased sympathetic activity could be observed in patients with end-stage renal disease, and also in patients with partially preserved renal function in polycystic kidney disease, or after renal transplant [44–46], as we demonstrated earlier in a cross-sectional study and these changes could be followed by HRR [16].

Our data show that in some of the patients with IgA nephropathy with impaired renal function (CKD stage 2–4) has autonomic dysfunction, which may be indicated by HRR reduction. Additionally, we assume that the effect of kidney damage on the increased sympathetic activity may be more important than other standard CV risk factors.

We examined several factors that could affect the CV status in patients with IgA nephropathy. We demonstrated that traditional metabolic risk factors (i.e. hypertension, carbohydrate metabolic disorders, lipidaemia and obesity) had effects on HRR, but the closest relationship is between HRR and eGFR.

In IgA nephropathy patients with metabolic syndrome exhibited significantly lower HRR and suffered more endpoints than those without metabolic syndrome. An increasing number of the metabolic syndrome components had led to greater HRR reduction in a longitudinal study of Kizilbash et al. [47]. We had similar findings, in addition, we also observed that the incidence of the primary endpoints was higher with the increased number of the metabolic syndrome components, indicating that early therapy and interventions for the correction of the metabolic parameters may be important in IgA nephropathy.

According to our multivariate regression analysis model in patients with IgA nephropathy reduced GFR may play a more important role in the development of sympathetic hyperactivity than DM or other risk factors examined. In the lower HRR group there was higher incidence of primary endpoints and diastolic dysfunction, suggesting that sympathetic hyperactivity could contribute to abnormal remodelling in the heart, and possibly in the kidneys as well, which may be responsible for the higher rate of CV and renal events. The question is how this sympathetic hyperactivity can be influenced most? Theoretically, the use of renin-angiotensin-aldosterone system inhibitors, and the use of sympathetic adrenergic blocking agents might be beneficial. Klein et al. reported that enalapril and losartan could decrease sympathetic hyperactivity in patients with CKD [48]. In the lower HRR group of IgA nephropathy patients ACE-inhibitors and ARBs were administered in higher proportions, thus RAAS blocker therapy alone could not have prevented the occurrence of primary and secondary endpoints. The use of beta blockers was comparable between the two groups. RAAS blocker and beta blocker therapy did not change during follow-up period.

The review published by Trimarchi and Coppo this year highlights the role of the development of microangiopathic / thrombotic microangiopathic (MA / TMA) lesions in the background of deteriorating renal function in patients with IgA nephropathy [49]. The role of these microangiopathic lesions may also arises in our patients, but this has not been investigated in our present study.

Kirkman et al. observed that aerobic exercise improved microvascular function and maintained conduit artery function, thus they concluded that it should be considered as an adjunct therapy to reduce CVD risk and progression of kidney failure in CKD [50]. Of the non-pharmacological intervention, regular physical activity could have a protective effect on both HRR and outcomes. Nevertheless, we cannot draw any conclusion regarding the effects of different treatments on the autonomic dysfunction in CKD patients from our earlier cross-sectional study or this current follow-up study. In further, controlled, large scale, randomized studies are needed to address this question.

### Limitation of the study

There were limiting factors in our study. Although the treadmill stress test is a simple, widely used method for measuring HRR, there are significant differences in the HRR threshold values in the literature. Protocols in the length of the recovery phase could also be different. Coronary angiography for diagnostic purposes of CAD was not performed, resulting in decreased sensitivity and specificity of CAD diagnosis. We could not find significant relationship in IgA nephropathy between CAD, functional capacity and HRR, probably due to the relatively small numbers of patients and shorter follow-up time. We studied only one type of kidney disease; however, we believe that this is the strength of the study, as the patient population was homogeneous.

The renal function was estimated, not measured, although the use of eGFR is widely accepted in the literature to describe renal function. Evaluation of the results may be weakened by the low number of cases. The follow-up time may have not been extended enough to determine differences in the CV event rate.

Although, it helped to highlight the fact that reduced HRR in response to treadmill exercise could predicted CV and renal outcomes in CKD preceding the onset of target organ damage.

## Conclusion

In our study, we showed that impaired renal function is an independent risk factor for reduced HRR. HRR has a prognostic value in IgAN for predicting CV morbidity and mortality. Nonetheless, long-term studies are necessary to confirm our findings, as well as to determine whether specific drug therapy could alter HRR and reduce CV risk in these patients.

## Data Availability

The datasets used and analysed during the current study are available from the corresponding author on reasonable request.

## References

[1] Myers J, Prakash M, Froelicher V (2002). Exercise capacity and mortality among men referred for exercise testing. N Engl J Med.

[2] Jouven X, Zureik M, Desnos M (2000). Long-term outcome in asymptomatic men with exercise-induced premature ventricular depolarizations. N Engl J Med.

[3] Arai Y, Saul JP, Albrecht P (1989). Modulation of cardiac autonomic activity during, and immediately after exercise. Am J Phys.

[4] Imai K, Sato H, Hori M (1994). Vagally mediated heart rate recovery after exercise is accelerated in athletes but blunted in patients with chronic heart failure. J Am Coll Cardiol.

[5] Buchheit M, Gindre C (2006). Cardiac parasympathetic regulation: respective associations with cardiorespiratory fitness and training load. Am J Physiol Heart Circ Physiol.

[6] Shetler K, Marcus R, Froelicher VF (2001). Heart rate recovery: validation and methodologic issues. J Am Coll Cardiol.

[7] GBD Chronic Kidney Disease Collaboration (2020). Global, regional, and national burden of chronic kidney disease, 1990–2017: a systematic analysis for the global burden of disease study 2017. Lancet.

[8] Floege J, Freehally J (2000). IgA nephropathy: recent developments. J Am Soc Nephrol.

[9] Cole CR, Blackstone EH, Pashkow FJ (1999). Heart-rate recovery immediately after exercise as a predictor of mortality. N Engl J Med.

[10] Vivekananthan DP, Blackstone EH, Pothier CE (2003). Heart rate recovery after exercise is a predictor of mortality, independent of the angiographic severity of coronary disease. J Am Coll Cardiol.

[11] Chen MS, Blackstone EH, Pothier CE (2004). Heart rate recovery and impact of myocardial revascularization on long-term mortality. Circulation.

[12] Jouven X, Empana JP, Schwartz PJ (2005). Heart-rate profile during exercise as a predictor of sudden death. N Engl J Med.

[13] Lipinski MJ, Vetrovec GW, Gorelik D (2005). The importance of heart rate recovery in patients with heart failure or left ventricular systolic dysfunction. J Card Fail.

[14] Shlipak MG, Fried LF, Crump C (2002). Cardiovascular disease risk status in elderly persons with renal insufficiency. Kidney Int.

[15] Vanholder R, Massy Z, Argiles A (2005). For the European uremic toxin work group (EuTox). Chronic kidney disease as cause of cardiovascular morbidity and mortality. Nephrol Dial Transplant.

[16] Késői I, Sági B, Vas T (2010). Heart rate recovery after exercise is associated with renal function in patients with a homogenous chronic renal disease. Nephrol Dial Transplant.

[17] Spies C, Otte C, Kanaya A (2005). Association of metabolic syndrome with exercise capacity and heart rate recovery in patients with coronary heart disease in the heart and soul study. Am J Cardiol.

[18] Turgut D, Yenigün EC, Kundi H, Özkayar N, Dede F (2020). Subclinical cardiovascular risk factors in chronic kidney disease: abnormal heart rate recovery. Turk J Nephrol.

[19] Grundy SM, Brewer HB, Cleeman JJ (2004). Definition of metabolic syndrome: report of the National Heart, lung and blood institute/ American Heart Association conference on scientific issues related to definition. Circulation.

[20] American College of Cardiology/American Heart Association Task Force on Practice Guidelines (2002). ACC/AHA 2002 Guideline update for exercise testing: summary article. A report of the American College of Cardiology/American Heart Association Task Force on Practice Guidelines (Committee to Update the 1997 Exercise testing guidelines). Circulation.

[21] von Känel R, Barth J, Kohls S (2009). Heart rate recovery after exercise in chronic heart failure: role of vital exhaustion and type D personality. J Cardiol.

[22] do Prado DL, Gualano B, Miossi R *et al.* Abnormal chronotropic reserve and heart rate recovery in patients with SLE: a case-control study. Lupus. 2011; 20: 717–720. doi: 10.1177/0961203310397081. 10.1177/0961203310397081 21596946

[23] Kaya EB, Yorgun H, Akdogan A (2009). Heart-rate recovery index is impaired in Behçet’s disease. Tex Heart Inst J.

[24] Singh JP, Larson MG, O’Donnell CJ (2000). Association of hyperglycemia with reduced heart rate variability (the Framingham heart study). Am J Cardiol.

[25] Lahiri MK, Kannankeril PJ, Goldberger JJ (2008). Assessment of autonomic function in cardiovascular disease: physiological basis and prognostic implications. J Am Coll Cardiol.

[26] Cole CR, Foody JM, Blackstone EH (2000). Heart rate recovery after submaximal exercise testing as a predictor of mortality in a cardiovascular healthy cohort. Ann Intern Med.

[27] Mora S, Redberg RF, Cui Y (2003). Ability of exercise testing to predict cardiovascular and all-cause death in asymptomatic women: a 20-year follow-up of the lipid research clinics prevalence study. JAMA..

[28] Cheng YJ, Lauer MS, Earnest CP (2003). Heart rate recovery following maximal exercise testing as a predictor of cardiovascular disease and all-cause mortality in men with diabetes. Diabetes Care.

[29] Jae SY, Carnethon MR, Heffernan KS (2008). Slow heart rate recovery after exercise is associated with carotid atherosclerosis. Atherosclerosis..

[30] Huang PH, Leu HB, Chen JW (2004). Usefulness of attenuated heart rate recovery immediately after exercise to predict endothelial dysfunction in patients with suspected coronary artery disease. Am J Cardiol.

[31] Paisley KE, Beaman M, Tooke JE (2003). Endothelial dysfunction and inflammation in asymptomatic proteinuria. Kidney Int.

[32] Ozkayar N, Akyel S, Dede F (2015). Evaluation of heart rate recovery in patients with primary nephrotic syndrome. HIPPOKRATIA.

[33] Qiu S, Cai X, Sun Z (2017). Heart rate recovery and risk of cardiovascular events and all-cause mortality: a Meta-analysis of prospective cohort studies. J Am Heart Assoc.

[34] Kiba T (2004). Relationships between the autonomic nervous system and the pancreas including regulation of regeneration and apoptosis: recent developments. Pancreas..

[35] Campos C (2012). Chronic hyperglycemia and glucose toxicity: pathology and clinical sequelae. Postgrad Med.

[36] Hevener AL, Febbraio MA, Stock Conference Working G. The 2009 Stock conference report: inflammation, obesity and metabolic disease. Obes Rev. 2010(11):635–44. 10.1111/j.1467-789X.2009.00691.x. 10.1111/j.1467-789X.2009.00691.x 20002885

[37] Davel AP, Wenceslau CF, Akamine EH (2011). Endothelial dysfunction in cardiovascular and endocrine-metabolic diseases: an update. Braz J Med Biol Res.

[38] Lauer MS (2009). Autonomic function and prognosis. Cleve Clin J Med.

[39] Carreira MAMQ, Nogueira AB, Pena FM (2015). Detection of autonomic dysfunction in hemodialysis patients using the exercise treadmill test: the role of the chronotropic index, heart rate recovery, and R-R variability. PLoS One.

[40] Ie EHY, Zietse R (2006). Evaluation of cardiac function in the dialysis patient—a primer for the non-expert. Nephrol Dial Transplant.

[41] Messias LR, Carreira MAMQ, Miranda SMR (2011). Relationship between cardiac adrenergic image and exercise testing in heart failure. Arq Bras Cardiol.

[42] Gayda M, Bourassa MG, Tardif JC (2012). Heart rate recovery after exercise and long-term prognosis in patients with coronary artery disease. Can J Cardiol.

[43] McManus D, Shlipak M, Ix JH (2007). Association of cystatin C with poor exercise capacity and heart rate recovery: data from the heart and soul study. Am J Kidney Dis.

[44] Vink EE, de Jager RL, Blankestijn PS (2013). Sympathetic hyperactivity in chronic kidney disease: pathophysiology and (new) treatment options. Curr Hypertens Rep.

[45] Converse RL, Jacobsen TN, Toto RD (1992). Sympathetic overactivity in patients with chronic renal failure. N Engl J Med.

[46] Klein IHH, Ligtenberg G, Oey PL (2001). Sympathetic activity is increased in polycystic kidney disease and is associated with hypertension. J Am Soc Nephrol.

[47] Kizilbash MA, Carnethon MR, Chan C (2006). The temporal relationship between heart rate recovery immediately after exercise and the metabolic syndrome: the CARDIA study. Eur Heart J.

[48] Klein IH, Ligtenberg G, Oey PL (2003). Enalapril and losartan reduce sympathetic hyperactivity in patients with chronic renal failure. J Am Soc Nephrol.

[49] Trimarchi H, Coppo R (2021). Glomerular endothelial activation, C4d deposits and microangiopathy in immunoglobulin a nephropathy. Nephrol Dial Transplant.

[50] Kirkman DL, Ramick MG, Muth BJ, et al. Aerobic exercise improved microvascular function and maintained conduit artery function and should be considered as an adjunct therapy to reduce CVD risk in CKD. Am J Phisiol Renal Phisiol. 2019. 10.1152/ajprenal.00539.2018.

